# A Quantum Chemical Investigation into the Molecular Mechanism of the Atmospheric Reactions of Chemi-Ions with Nitrogen and Nitrogen Oxides

**DOI:** 10.3390/e24091257

**Published:** 2022-09-07

**Authors:** Rehin Sulay, Anandhu Krishnan, Balasubramoniam Muralikrishna, Sudheesh Devadas, Chandralekha Rajalakshmi, Jintumol Mathew, Vibin Ipe Thomas

**Affiliations:** 1 Department of Chemistry, CMS College Kottayam (Autonomous), Kottayam 686001, Kerala, India; 2 Institute for Integrated Programmes and Research in Basic Sciences, Mahatma Gandhi University, Priyadarsini Hills P.O., Kottayam 686560, Kerala, India

**Keywords:** chemi-ion, nitrogen oxides, computational modeling, activation energy, transition state

## Abstract

Nitrogen oxides and chemi-ions are atmospheric pollutants with considerable aeronomic interest. These toxicants can react with each other, producing various ionic species and highly reactive by-products that play a crucial role in aerosol clustering and mediate several important atmospheric reactions. Understanding the chemical reactivity of these pollutants can provide essential information for controlling their excess emission into the atmosphere. Computational modeling and electronic structure studies help in predicting the structure, reactivity, and thermodynamics of transient atmospheric chemical species and can guide experimental research by providing vital mechanistic insights and data. In the present study, a computational investigation into the mechanisms of the binary associative reactions between negative ions: O_2_^−^ and O_3_^−^ with NO, NO_2_, and N_2_ was conducted using the Coupled-Cluster Singles and Doubles (CCSD) theory. Five model reactions between N_2_/NO_x_ with O_n_^−^ (*n* = 2, 3) were considered in this work. Our calculations revealed that reactions (2) and (5) are two sequential processes involving intermediates, and all others occur in a concerted manner by direct transitions from the reactants to the products, with no isolable intermediates proceeding via single non-planar transition states. Our study revealed that the higher activation barrier required for the formation of NO_3_^−^ (2) as compared to NO_2_^−^ (1) could be the reason for the excess formation of NO_2_^−^ ions over NO_3_^−^ ions in the atmosphere. Further, all the investigated reactions except (5) are found to be feasible at room temperature. The energy required to break N-N bonds in the N_2_ molecule justifies the high barrier for (5). The results obtained from the study are in close agreement with the available experimental data. Moreover, the data from the study can be utilized for the evaluation of experiments and model predictions pertaining to NOx oxidation and molecular modeling of the gas-phase chemistry of pollutants/nucleation precursors formed in the Earth’s atmosphere and aircraft engines.

## 1. Introduction

The chemistry of pollutants in the different regions of the atmosphere is of considerable significance. Some of the major atmospheric pollutants include nitrogen oxides (NOx), sulfur oxides (SOx), carbon monoxide (CO), particulate matter (PM), and more [1,2]. Reactions of these contaminants with varied chemical species present in the atmosphere pose severe threats to the biosphere [3,4]. For instance, the atmosphere’s reaction chemistry of nitrogen oxides (NOx) is of significant interest because of its environmental impact. Nitrogen oxides are toxic air pollutants produced mainly by the reaction between nitrogen and oxygen/ozone at elevated temperatures. One of the sources of NOx emission in the atmosphere, particularly in the stratospheric and tropospheric regions, is the air transport system [5,6,7]. Combustion products from the aircraft exhausts are discharged directly into the upper and lower stratosphere regions, leading to stratospheric ozone depletion [8,9,10]. Eventually, this accelerates the greenhouse effect [11] and increases the quantity of harmful ultraviolet radiation from the sun reaching the earth’s surface [12]. In addition, NO_x_ can react with moisture, ammonia, or ozone to form acids such as nitric acid and subsequently become deposited on the earth’s surface as acid rain [13]. These wet or dry depositions (with high hydrogen ion concentration) can have destructive effects on the soil [14], aquatic life [15], natural vegetation [16], and human health [17]. Molecular-level insights into these chemical transformations can essentially aid in revealing their impact on global climate change and atmospheric pollution [18,19].

One of the less explored sets of reactions with critical detrimental effects on the environment is between nitrogen oxides (NO_x_) and different chemi-ions (CI) [20]. Chemi-ions, both positively and negatively charged, are essential constituents in the ionospheric region of the atmosphere [21,22,23]. They are formed by ion-molecule charge transfer reactions in the atmosphere [20,24] and as by-products in combustion processes in diesel engine exhaust systems [25,26,27]. HSO_4_^−^, NO_3_^−^, O^−^, O_2_^−^ and O_3_^−^ are a few of the foremost negative-ion species in the atmosphere. The formation and evolution of chemi-ions in aircraft engines and the atmosphere are well studied [26,28,29,30,31]. It is well known that chemi-ions, particularly negatively charged ions, play a significant role in the formation of volatile aerosol particles [28,32,33], which pose adverse effects on both human health [34] and the environment [35]. These negative ions could react with nitrogen and nitrogen oxides in the atmosphere and aircraft engines to generate ions that contribute to forming aerosol clusters [26,36]. Hence, it is necessary to understand the underlying mechanism behind these reactions to control their harmful effects. Though theoretical studies of reaction between sulphur oxides and chemi-ions are available [37], to the best of our knowledge and based on our literature survey, detailed computational studies of NO_x_-chemi-ion reactions are scarce. Fehsenfeld et al. [20] analyzed and studied the kinetics of negative ion reactions with NO_x_. They explored the associative detachment mechanism for the loss process of negative oxygen ions through laboratory experiments. These experimental data provide the basis for broadening the understanding of chemi-ion-NO_x_ interactions.

Owing to the complexity of these atmospheric reactions, it is often difficult to depict their fundamental reaction mechanisms with the most accuracy using only experimental studies. Moreover, the key intermediates involved in such reactions are mostly transient chemical species, which are often untraceable experimentally due to their rapid reactivity. Though early studies in atmospheric chemistry were driven mainly by experimental works, computational modeling and quantum chemical calculations emerged as powerful tools in atmospheric chemistry research over the last few years. Identifying the key intermediates and reaction pathways involved is crucial for obtaining molecular-level mechanistic insights into chemical reactions. With the advent of computational power, the reactivity of diverse chemical species in the atmosphere could be computationally studied for extracting relevant structural, mechanistic, and thermodynamic data with less time and cost [38]. Computational tools, therefore, complement experimental studies by creating electronic structure models, predicting and elucidating the complex mechanisms of chemical reactions that take place rapidly in the atmosphere, and identifying the possible reactive intermediates that are otherwise tedious and onerous to study using experiments [39]. The integration of experimental and theoretical studies can thus aggregate the essential information required for understanding atmospheric chemical reactions and thereby aid in converting the knowledge to tackle the possible environmental plights.

In the present study, we have computationally investigated the mechanism involved in the associative reaction of nitrogen and nitrogen oxides with negative chemi-ions, such as O_2_^−^ and O_3_^−^, utilizing electronic structure calculations. O_2_^−^ and O_3_^−^ ions were chosen as the model chemi-ions because of their structural simplicity and similarity with neutral O_2_ and O_3_ molecules, whose reactions with NOx are well-studied [40,41]. We have utilized the end-atom approach to probe the most favorable reaction pathway [42]. We have considered the following set of reactions as our model systems:

**I.** NO + O_3_^− →^ NO_2_^−^ + O_2_ (1)

**II.** NO + O_3_^− →^ NO_3_^−^ + O (2)

**III.** NO_2_ + O_3_^− →^ NO_3_^−^ + O_2_ (3)

**IV.** NO + O_2_^- →^ NO_2_^-^ + O (4)

**V.** N_2_ + O_3_^− →^ NO_2_^-^+ NO (5)

A potential application for understanding the mechanisms of NOx-chemi-ion reactions is in the development of technologies that can safely remove both these toxicants in the form of useful chemicals. For example, the nitrate ion generated via reaction IV can easily become protonated to form nitric acid. If the nitric acid formed is released into the atmosphere, it could promote serious biological and environmental destruction [15,43,44,45]. However, nitric acid is an industrially important chemical [46,47]. It would be economically and environmentally beneficial to produce and store nitric acid by converting toxicants like NOx and chemi-ions from combustion exhaust. Since there will be a significant surge in air traffic in the coming years, controlling the nitrogen oxide concentration in the atmosphere and developing aircraft with minimal release of pollutants is a priority of the future [6]. In this context, a detailed understanding of the reaction mechanism between nitric oxides and chemi-ions, could aid the aviation industry in developing such technologies and designing environmentally friendly aircraft with reduced emissions of these pollutants [48].

Furthermore, reactions of these negative ions with minor atmospheric constituents like NO, NO_2_, and SO_2_ are imperative in aeronomy and radiation chemistry and serve as essential data on the energetics and mechanism of gas-phase reactions [49]. Since nitrogen is the most prevalent gas in the atmosphere and the reaction of SO_X_ with O_3_^−^ has already been investigated, our study is limited to the reaction mechanism of N_2_/NO_x_ with O_n_^−^ in the atmosphere [37]. To date, very limited information about the reaction mechanisms of chemiions with CO and PM have been reported. Their reaction channel should be studied further in the future.

In view of the expected significance of the title reactions in the atmosphere, this work proposes to elucidate the fundamental molecular mechanism of the reactions using the Couple Cluster theory. In addition, the study identifies the thermodynamically viable reaction channels and thereby determines the reaction’s feasibility of occurring in the atmosphere. The primary products of the reactions examined here are a number of extremely reactive and polar nitrogen oxide anions, which may either be neutralized through ion–ion recombination or may interact with various airborne molecules to form large cluster ions.

## 2. Computational Details

The geometries of all the reactants, transition states, and products involved in reactions I–V were optimized using the Coupled-Cluster Singles and Doubles (CCSD) theory [50], which is an efficient post-Hartree–Fock method. The CCSD method provided an adequate balance between computational cost and accuracy. The reliability of the CCSD method in studying atmospheric chemical reactions was evident from the results of the present study, which report close agreement of CCSD geometry optimizations and energy data with the available experimental values [40,41]. Single-point energy calculations were carried out by employing the Coupled-Cluster Singles Doubles, and Triples [51] (CCSD(T)) method with the basis set augmented correlation consistent polarized valence triple ζ (aug-CCPVTZ) [51,52] to obtain more reliable relative energies for the stationary points on the potential energy surface (PES).

We found that the CCSD/6-311+G(d) theory and basis sets were largely sufficient for the purpose and aims of our work. The stationary points obtained were characterized as minima or a transition state by vibrational frequency analysis. The minima is the stationary point with no imaginary frequency, whereas the stationary point having only one imaginary frequency is classified as a transition state [53]. Further, the transition states were confirmed by the intrinsic reaction coordinate (IRC) [53] calculation at CCSD/6-311+G(d) level. The zero-point energy (ZPE), activation energy, and the change in the vibrational degree of freedom in moving from reactant to product were calculated. All calculations were carried out using Gaussian 09 [54] software.

## 3. Results and Discussion

**Scheme 1:**
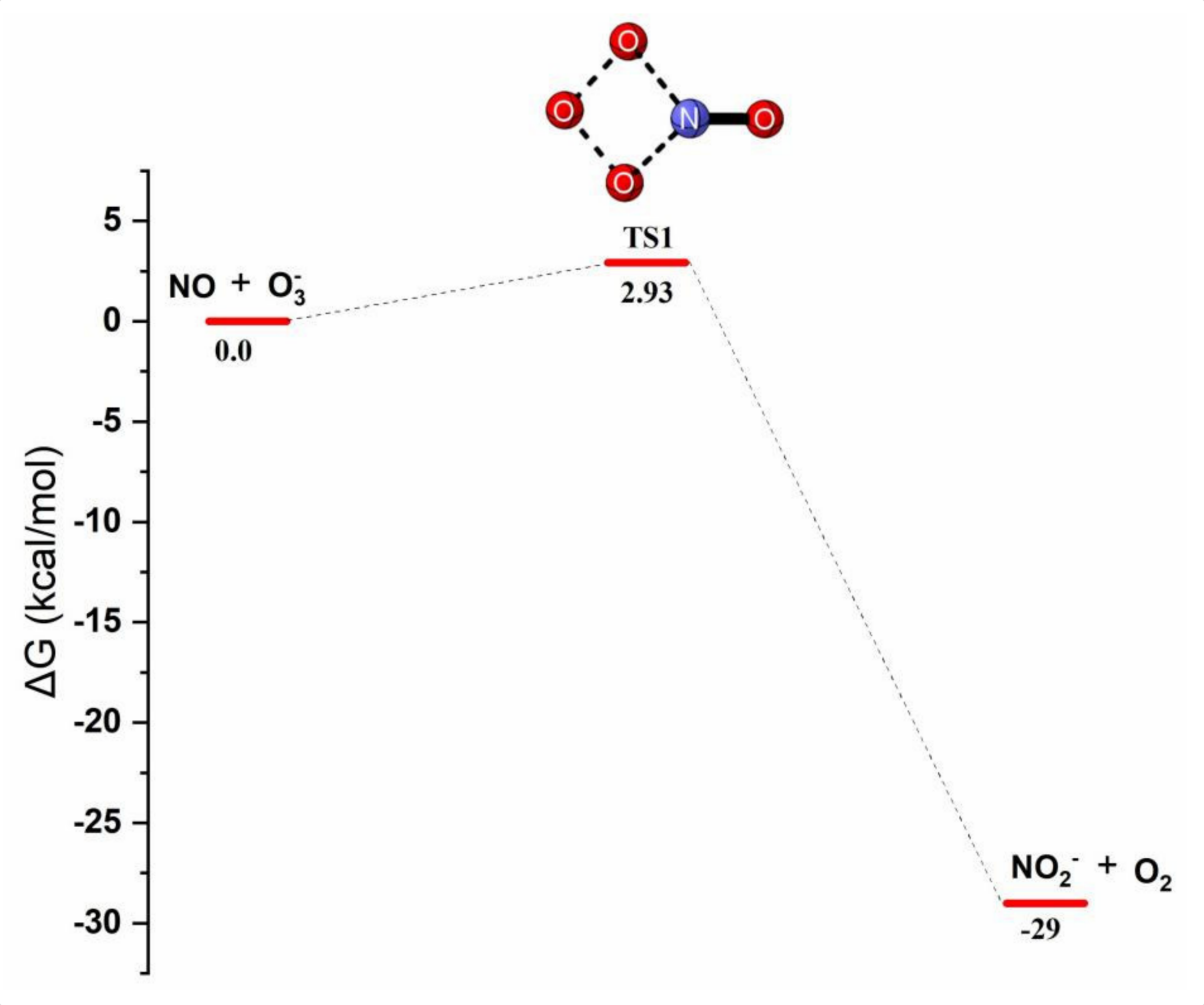
Energy profile for theNO + O_3_^− →^ NO_2_^−^ + O_2_ reaction system CCSD(T)/aug-cc-pVTZ level. TS1 represents the saddle point. Relative energies with respect to the reactants are given in kcal/mol.

**Scheme 2:**
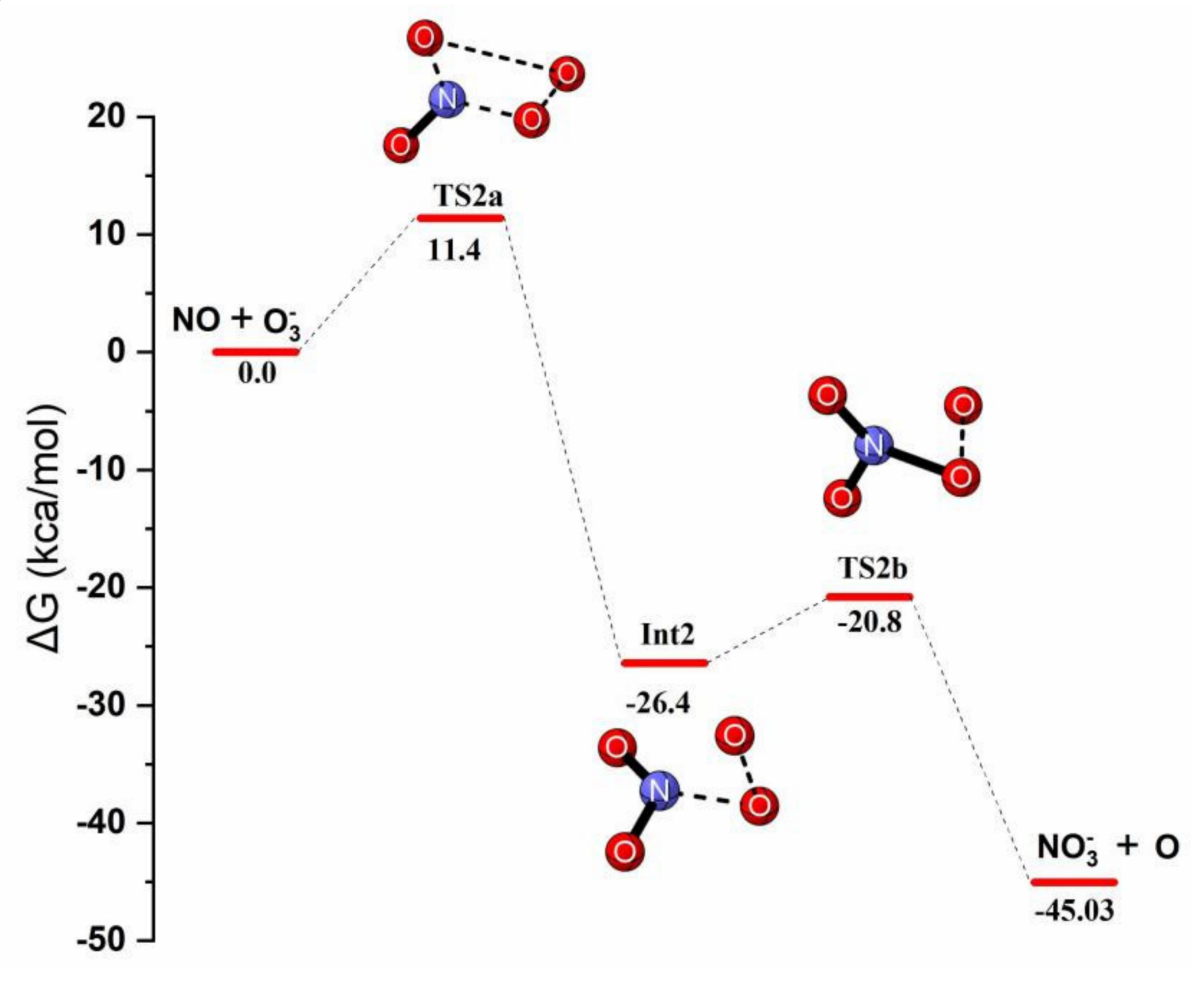
Energy profile for the NO + O_3_^− →^ NO_3_^−^ + Oreaction system CCSD(T)/aug-cc-pVTZ level. TS2a and TS2b represent the saddle point. Relative energies with respect to the reactants are given in kcal/mol.

**Scheme 3:**
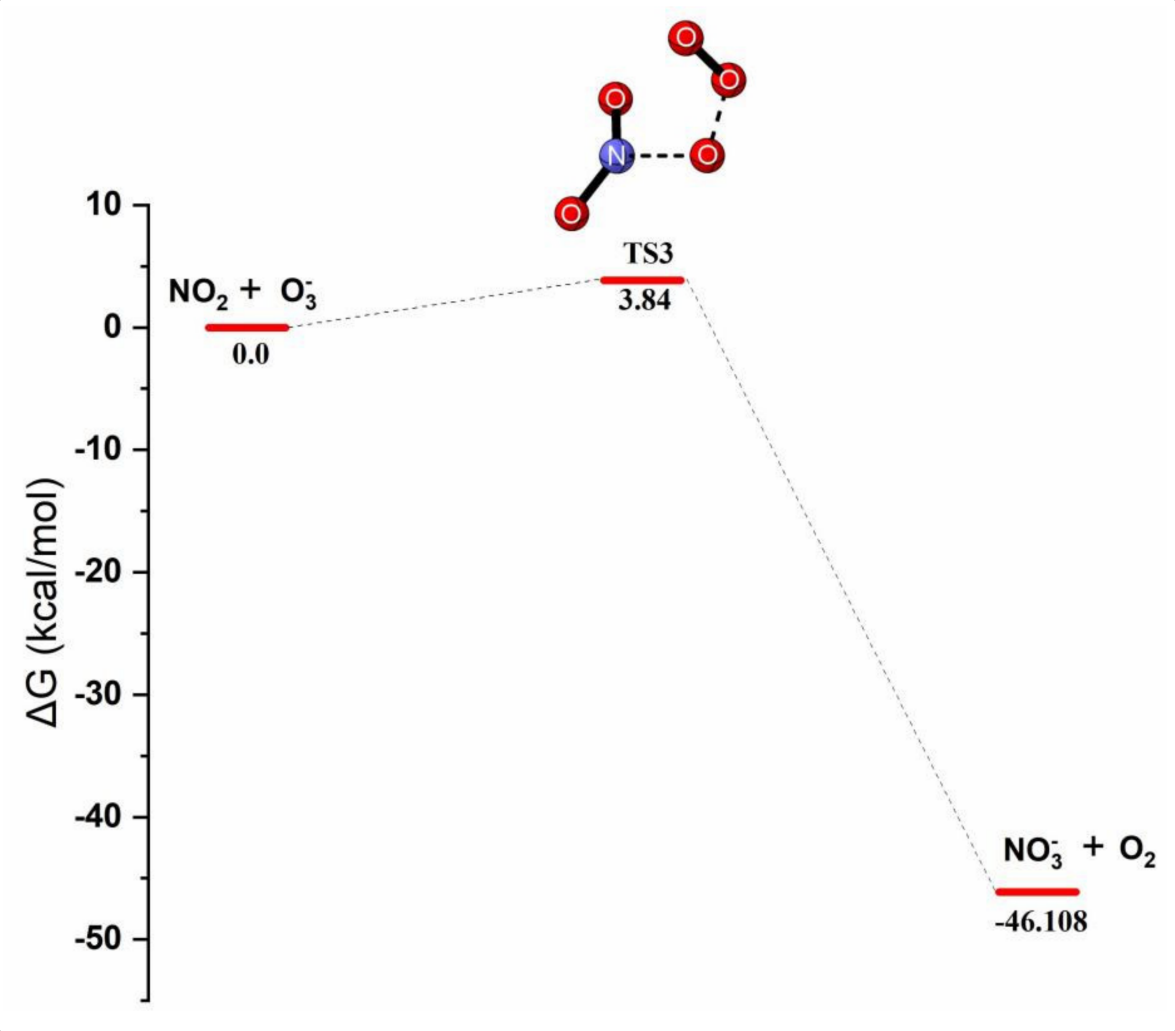
Energy profile for the NO_2_ + O_3_^− →^ NO_3_^−^ + O_2_ reaction system at CCSD(T)/aug-cc-pVTZ level. TS3 represents the saddle point. Relative energies with respect to the reactants are given in kcal/mol.

**Scheme 4:**
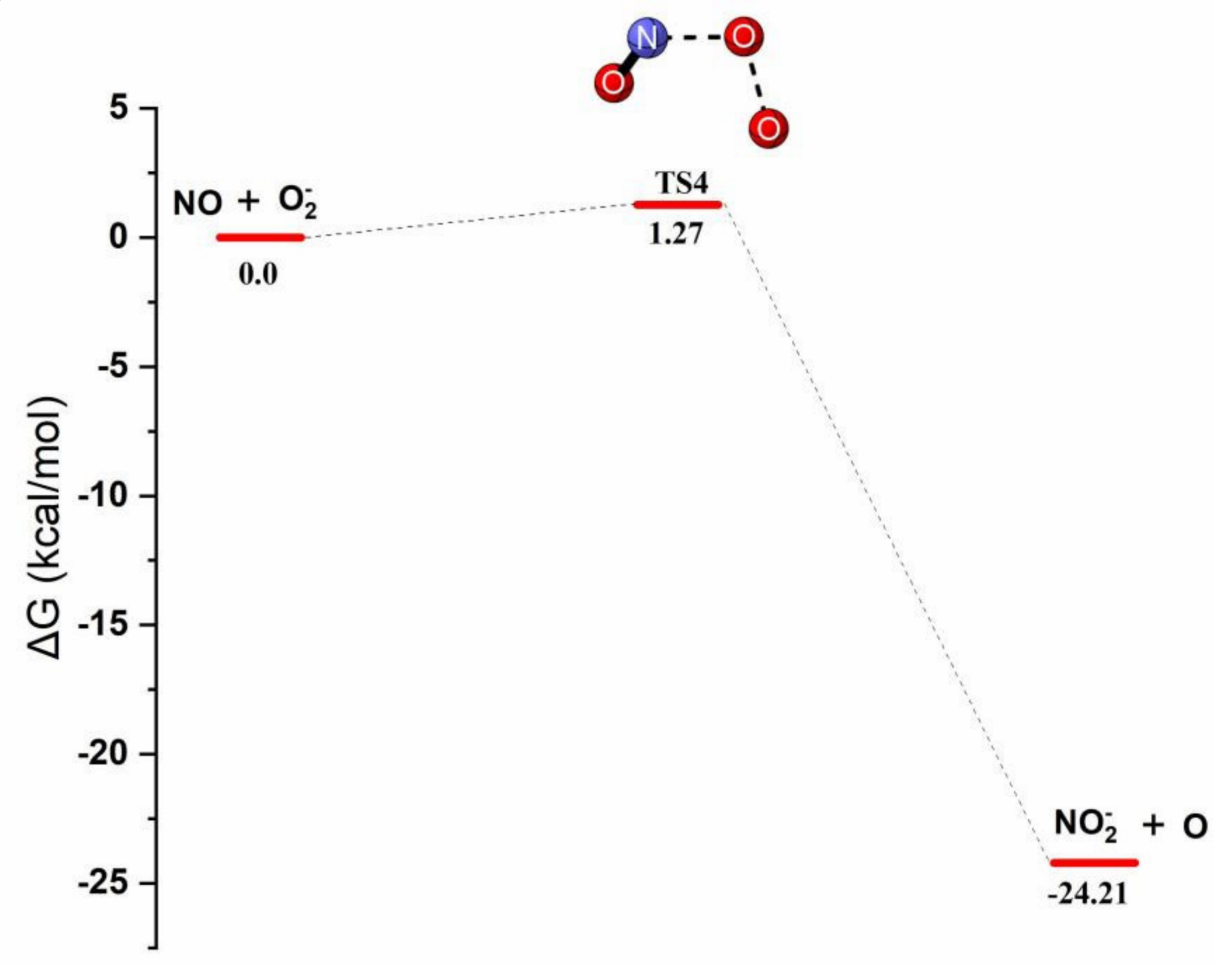
Energy profile for the NO + O_2_^− →^ NO_2_^−^ + O_2_ reaction system at CCSD(T)/aug-cc-pVTZ level. TS4 represents the saddle point. Relative energies with respect to the reactants are given in kcal/mol.

**Scheme 5:**
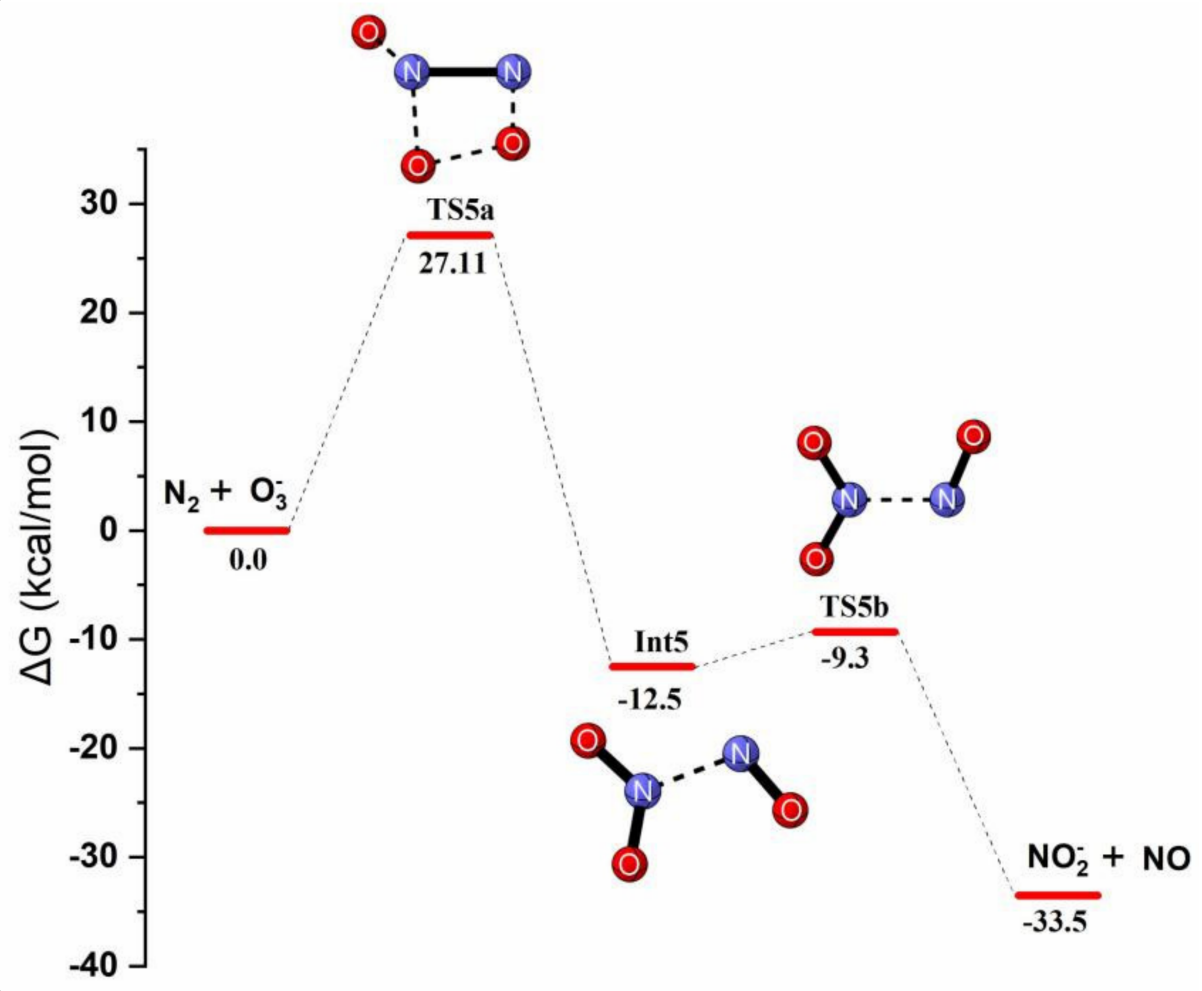
Energy profile for the N_2_ + O_3_^− →^ NO_2_^−^ + NO reaction system at CCSD(T)/aug-cc-pVTZ level. TS5a and TS5b represent the saddle point. Relative energies with respect to the reactants are given in kcal/mol.

The geometrical parameters of the stationary structures involved in the reactions I–V are listed in Table 1. The reaction pathway [53] for the reaction between negatively charged O_n_^−^ and N_2_/NO_x_ was characterized by exploring the potential energy surface (PES) method. The reactants, products, and saddle points involved in the reaction were optimized at the CCSD/6-311+G(d) level of theory. Further single-point energy calculations have been carried out with the CCSD(T)/aug-ccPVTZ level of theory. The energy profiles for the reaction of O_n_^−^ and N_2_/NO_x_ at the CCSD(T)//CCSD are depicted in Scheme 1, Scheme 2, Scheme 3, Scheme 4 and Scheme 5.

The optimized geometrical parameters for the reactants and products, as shown in Table 1 were found to be in agreement with the experimental values reported previously [40]. In the case of the NO molecule, the computationally calculated N-O bond length exactly matches the experimental value. In NO_2_, NO_2_^−^, and NO_3_^−^, the deviation of computationally calculated values of N-O bond from the experimental values are −0.009, −0.004 and −0.002, respectively. Furthermore, the difference is +0.005 Å, −0.001 Å and −0.003 Å for O-O bond in O_3_^−^, O_2_^−^ and O_2_, respectively. For the N_2_ molecule, a difference of 0.005 Å was observed for the N-N inter-atomic bond distance compared with the experimental values. As shown in Table 1, the largest difference between the calculated and experimental bond lengths is observed in the case of a neutral NO_2_ molecule, where the calculated N-O bond length deviates from the experimental value by 0.009 Å. Furthermore, the bond angles obtained from our calculations also agreed well with the experimental values. In the case of NO_3_^−^, the computed bond lengths and angles are consistent regardless of the basis set used and always exhibited *D_3h_* symmetry with 120° bond angle. At 6-311+G(d), NO_2_ and O_3_^−^ show a zero-bond angle gradient, whereas NO_2_^−^ shows a slight difference of 0.1° from the experimental values. The maximum deviation of 0.9° is observed for O_3_^-^ molecule at 6-31G(d) basis set.

To further scrutinize the reliability of the CCSD theory, we have conducted a vibrational analysis of all the reactants and products involved in the reactions (Table 2). The vibrational frequencies of all the reactants and products involved in reactions are listed in Table 2. The vibrational frequency obtained has been compared with the available experimental values, and from the analysis, the anharmonic frequencies of all species were found to be mostly consistent with experimental values. The results obtained for NO, NO_3_^−^, N_2_ and O_2_ are analogous to the experimental values, with deviations lying within ±100 cm^−1^. From the previous studies, neutral NO_3_ is found to be highly symmetrical and belongs to the *D*_3*h*_ point group at the CCSD level. The experimental studies clearly show that the NO_3_ belongs to the point group *D*_3*h*_. The six vibrational modes of NO_3_^−^ spans over A1′ +A2′ +E′ +A1″ +A2″+ E″ vibrations, representing a *D*_3*h*_ point group. The experimental evidence also indicates planar *D*_3*h*_ for NO_3_^−^ (webbook.nist.gov) (accesed on 12 March 2021). We observed that the calculations with the 6-311+g(d) and 6-31g(d) basis sets gave comparatively smaller variations from the experimental value. Surprisingly, 6-311g(d) exhibited larger variations than 6-31g(d) despite its theoretical supremacy over 6-31g(d). (Mode of vibrations are shown in Table S1 in the supplementary information).

The calibration of basis sets with bond parameters and vibrational frequencies shows that the more accurate result is obtained for 6-311+G(d) rather than the 6-31G(d) and 6-311G(d) basis sets. The subsequent molecular mechanistic calculations were conducted using the 6-311+G(d) basis set based on these observations.

### Reaction Mechanism

To characterize all the stationary points and roughly locate the transition states involved in the reactions, potential energy surface (PES) scans were performed initially at the CCSD level for all the transition state structures. The geometries obtained at the critical point were optimized using the Berny optimization algorithm, and the corresponding vibrational frequencies were calculated at the CCSD level. The potential energy surface scan showed that out of the five reactions considered in this study, two reactions proceed via a sequential manner involving intermediate species, while the other three reactions follow a concerted mechanism devoid of any intermediates. The total electronic energies and zero-point energies are provided in Table 3. In fact, we found that the total energy and zero-point energy values are accepted in the case of NO, NO_2_ and O_2_, as in the previous work reported [41].

**1.** NO + O_3_^− →^ NO_2_^−^ + O_2_

**Figure 1:**
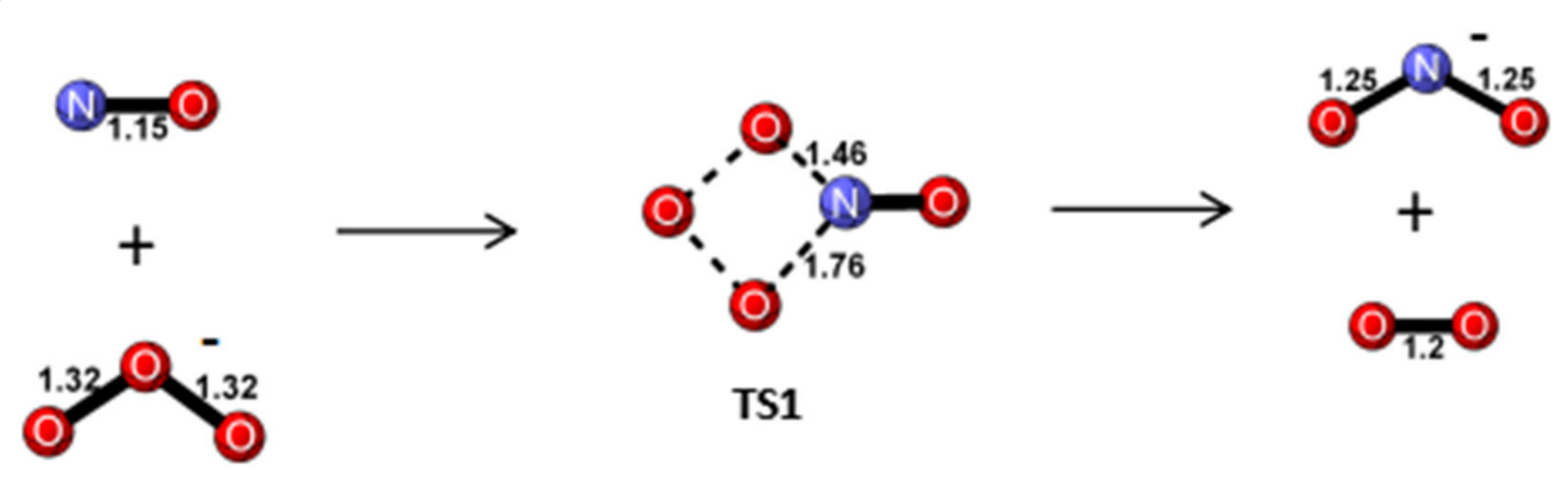
Optimized geometries of the reactants, transition state, and products for NO + O_3_^− →^ NO_2_^−^ + O_2_ reaction. Bond lengths are given in angstrom.

The proposed mechanism for the reaction between NO + O_3_^−^ to form NO_2_^−^ is presented in Figure 1. Here, the reaction is initiated by the interaction between the terminal oxygen in O_3_^−^ and the N atom in NO, leading to the transition state TS1, which then converts into the products NO_2_^−^ and O_2_. The potential energy hypersurface obtained for reaction I showed a critical point at an N-O bond distance of 1.73 Å. The Berny optimization leads to a four-centered transition state structure with only one imaginary frequency (−261.83 cm^−1^) for the stationary point. The corresponding N-O bond distance for the optimized transition state TS1 was found at 1.46 Å. TS1 has a *C*_1_ symmetry similar to the transition state in the reaction: NO + O_3_, and a non-planar structure reported earlier for the reaction: Cl + O_3_ [40]. On comparing the reactions of NO with O_3_ and O_3_^−^, it is understood that the reaction between NO and neutral ozone is sequential, involving two transition states and one intermediate. In contrast, the reaction of NO with charged ozone follows a concerted mechanism in which there is no intermediate present. From TS1, the dissociation of the O_2_ molecule, as well as the formation of NO_2_^−^ takes place simultaneously. 

The energy profile for reaction I calculated at the CCSD(T)/aug-cc-pVTZ level is shown in Scheme 1. The electronic energies and zero-point energies obtained from our calculations were validated by comparison with the results from previous studies on the NO-O_3_ system by Jaroszyńska-Wolińska [41] (Table 3), and we found that for NO, the total energy and zero-point energy values of −129.647 and 0.004 atomic units obtained from our calculations are authentic. Previous studies by Hwang et al. (Hwang and Mebel, 1998) have shown that the transition state obtained for the reaction of neutral ozone with NO and Cl are much closer to the reactant side, which implies that these reactions are exergonic in nature. Similarly, the transition state for the reaction between NO and charged ozone (O_3_^−^) shows only a small perturbation from the reactant side, indicating that this reaction is also exergonic. The calculated activation barrier for the reaction is 2.93 kcal/mol at CCSD(T)/aug-cc-pVTZ level of theory, confirming the feasibility of this reaction pathway.

**2.** NO + O_3_^− →^ NO_3_^−^ + O

**Figure 2:**
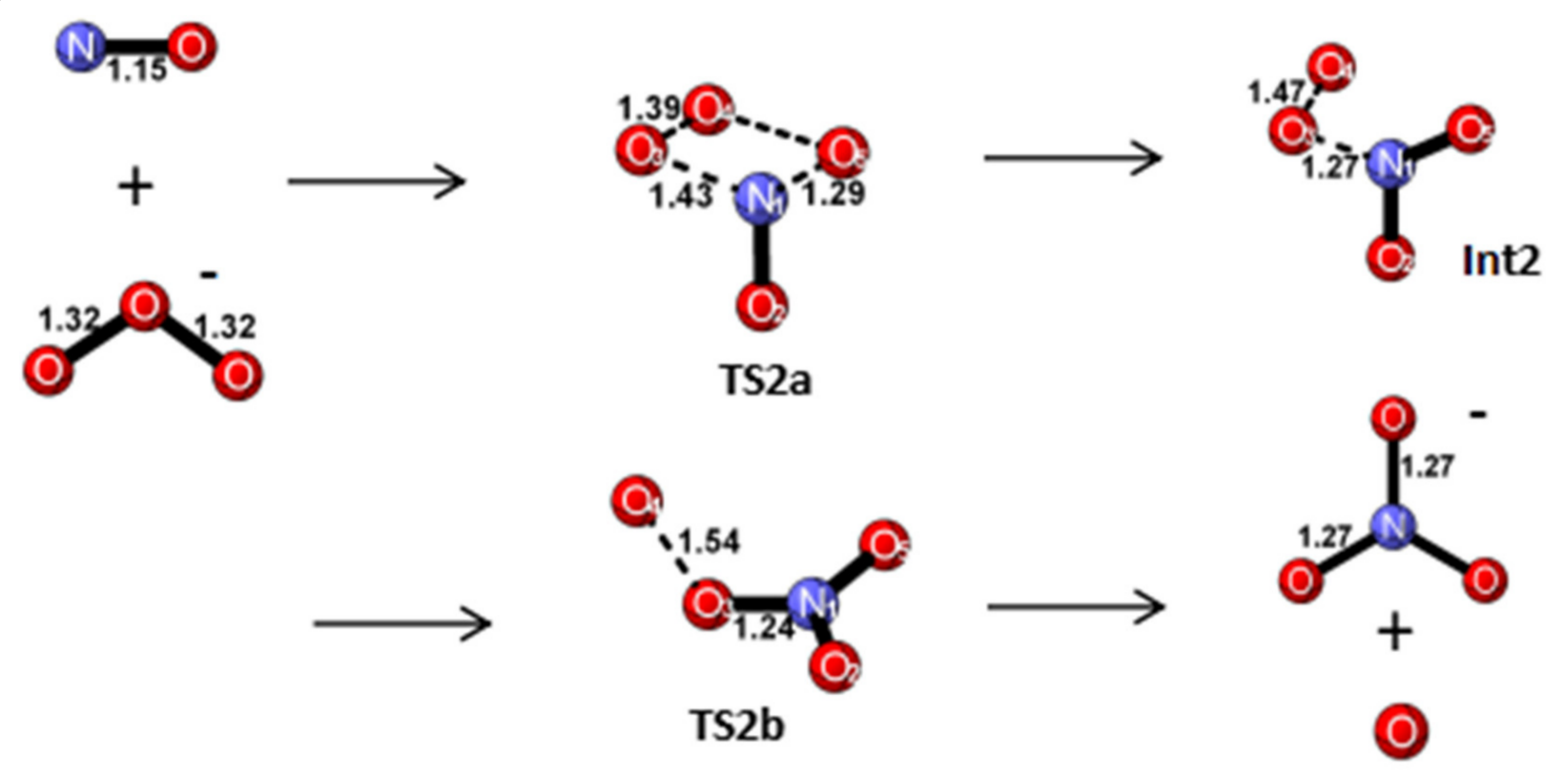
Optimized geometries of the reactants, transition state and products for the NO + O_3_^− →^ NO_3_^−^ + O reaction. Bond lengths are given in angstrom.

Another known reaction between NO and O_3_^-^ is the formation of NO_3_^−^ along with highly reactive atomic oxygen. The PES analysis of N-O bond length from 1.6 to 1.2 Å at CCSD/6-311+g(d) revealed that this reaction involves an intermediate (minima) and has two saddle points (maxima). The geometry at these points was optimized with the Berny algorithm to obtain the four-centred transition state TS2a with the C_2_ point group and another two-center transition state TS2b of C_s_ symmetry (Figure 2). Additional verification of these transition states was done through vibrational analysis of TS2a and TS2b, which gave only one imaginary frequency for each of them, −437.5 for TS2a and −132.8 for TS2b. The initial step of the reaction proceeds via TS2a. To reach TS2a, the two terminal oxygen atoms (O3 and O5) of O_3_^−^ come closer to the nitrogen forming an N1-O5 and N1-O3 bond length of 1.29 Å and 1.43Å, respectively. TS2a transforms to intermediate Int2, by the simultaneous formation of N1-O5 bond and the breakage of the O5-O4 bond. In non-planar Int2 the O4-O3 bond stretches from 1.39 to 1.47 Å and N1-O3 shortened to 1.27 Å from 1.43 Å. From Int2 the products are readily obtained through TS2b. The structure of TS2b has N1-O3 bond distance of 1.24 Å and O3-O4 distance of 1.54 Å. Thus, the O_3_^−^ associates with the NO molecule via terminal oxygen atoms. Here, the product NO_3_^−^ formed can combine with an H^+^ to form nitric acid. The CCSD(T)/aug-cc-pVTZ energy diagram for reaction II is given in Scheme 2. The activation energy for the initial reaction step is calculated to be 11.4 kcal/mol. The earlier experimental studies carried out by Fehsenfeld et al. [20] reveal that in the ionosphere, the production of NO_2_^−^ ions greatly exceeds the production of NO_3_^−^ ions. This observation can be validated through our calculations for the reaction between NO and O_3_^−^, which account that a higher activation barrier is required for the formation of NO_3_^−^ as compared to that for NO_2_^−^.

**3.** NO_2_ + O_3_^− →^ NO_3_^−^ + O_2_

**Figure 3:**
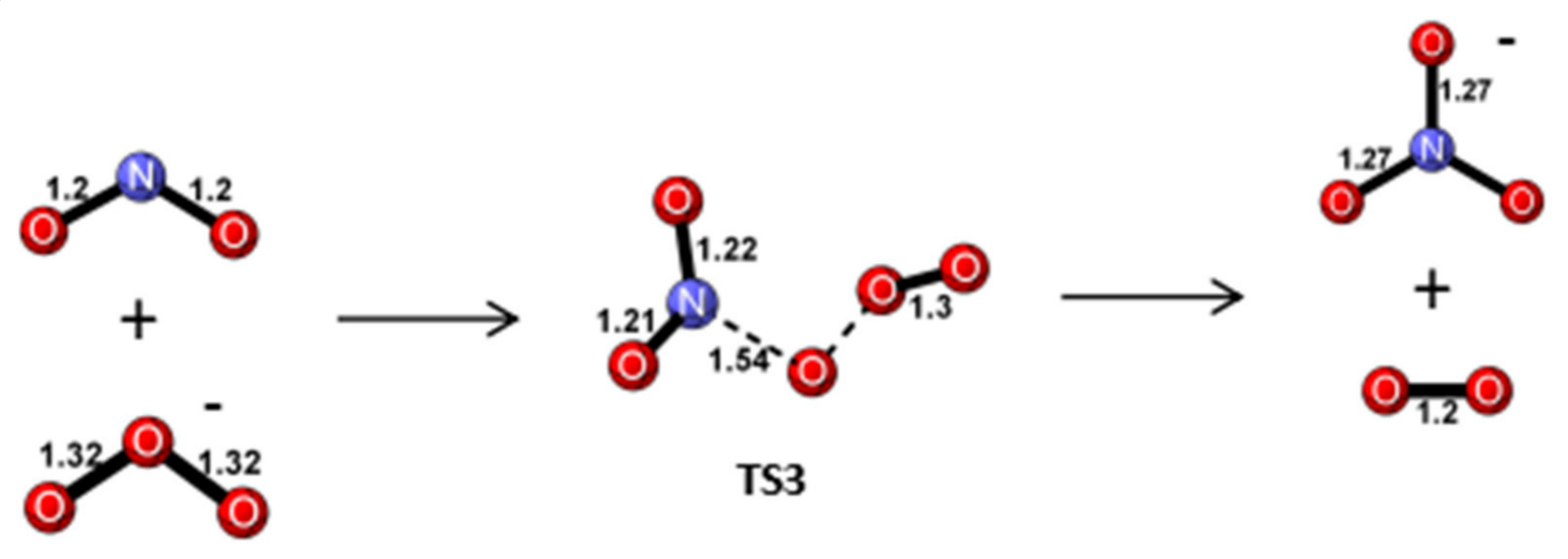
Optimized geometries of the reactants, transition state and products for the NO_2_ + O_3_^− →^ NO_3_^−^ + O_2_ reaction. Bond lengths are given in angstrom.

The potential energy scan for the reaction was carried out by varying the N-O bond distance from 1.0 Å to 2.0 Å, and a fine grid calculation between 1.4 Å to 1.8 Å was performed to locate the transition state. From the obtained potential energy hypersurface, a critical transition point was located at 1.6 Å. The geometry obtained at this point was optimized using the CCSD/6-311+G(d) level of theory. Figure 3 shows the structural parameters for the reactants, transition state and products involved in the reaction. The geometrical parameters computed for the transition state TS3, and reactants appear similar, which indicates that the saddle point for the reaction, as expected for an exergonic reaction, is located at the entrance of the reaction channel. The IRC calculation further indicates that the transition state connects the reactants to products. This confirms a direct reaction mechanism without any intermediates involved.

The first step in the reaction involves the attack of the terminal oxygen of O_3_^−^ on the N atom in nitrogen dioxide (NO_2_), as in the previous work carried out by Dupuis [59]. In the transition state TS3, the newly formed N-O bond distance is 1.54 Å. The transition state TS3 has a non-planar structure with C1 symmetry and an overall negative charge [56], similar to studies reported earlier for the reactions—NO + O_3_, NO_2_ + O_3_, Cl + O_3_ [40]. The imaginary frequency corresponding to the reaction coordinate of TS3 is −121.90 cm^−1^ at the CCSD/6-311+G(d) level. The previous work carried out by Garcia and Gill [40] shows that at the UMP2 level of theory, the reaction of neutral ozone with NO and NO_2_ proceeds as two-step reactions. However, when the single-reference higher correlated CCSD and QCISD methods were employed, only one transition state was optimized successfully while the second transition state always converged to the product. In the case of O_3_^−^ also the CCSD level of theory predicts that the reaction takes place in a concerted manner. The initial step and transition state of the NO_2_ + O_3_^−^ is quite similar to those of the NO_2_ + O_3_ reaction. The CCSD/6-311+G(d) energy diagram for reaction III is given in Scheme 3. The total energy and zero-point energy values of NO_2_ and O_2_ calculated at the 6-311+G(d) level of theory are closer to the values given in previous works [41]. Here, the activation energy required for the reaction is calculated to be 3.84 kcal/mol.

**4.** NO + O_2_^− →^ NO_2_^−^ + O

The reaction of superoxide (O_2_^−^) with nitrogen monoxide (NO) forms an important class of atmospheric reactions. PES analysis for the reaction showed only one critical point at N-O bond length of 1.54 Å. The optimization of the corresponding geometry shows only one imaginary vibrational frequency corresponding to a value of −434.9 cm^−1^. Here, the reaction of proceeding via the direct end-to-end atom approach where the HOMO of the superoxide combines with the LUMO of the NO leading to a transition state TS4 with an N-O bond distance 1.24 Å. The activated complex TS4 was expected to be linear in geometry. Still, the geometry optimization leads to a co-planar transition state structure in which the dihedral angle O-O-N-O corresponds to a value of 78 degrees with *C*_1_ symmetry. The reaction forms the highly stable atmospheric species NO_2_^−^ by directly dissociating an oxygen atom from the O_2_^−^ anion.

**Figure 4:**
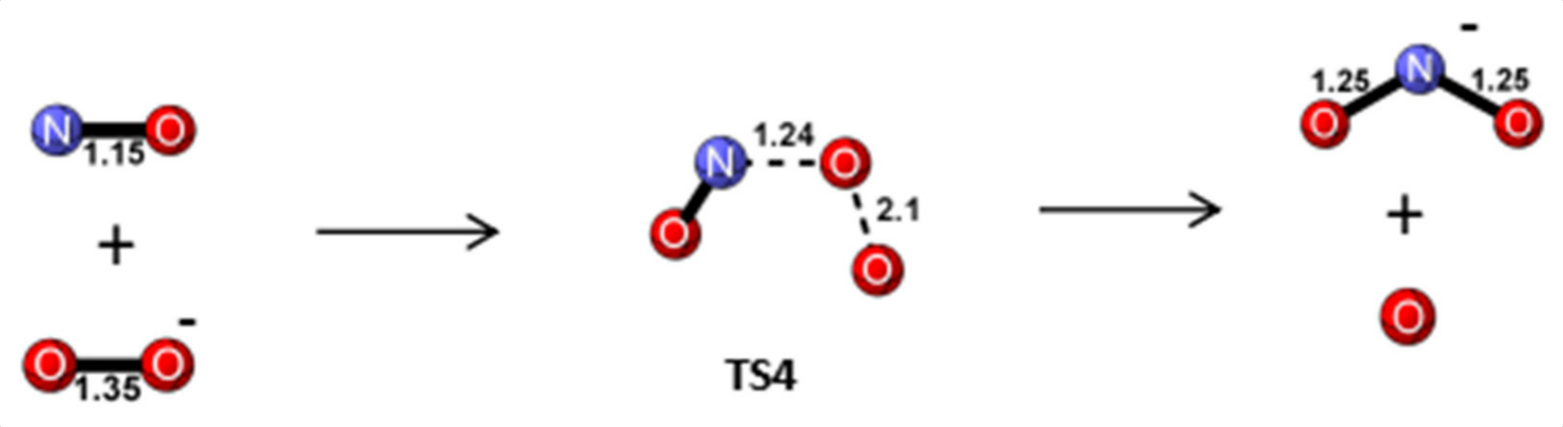
Optimized geometries of the reactants, transition state and products along NO + O_2_^− →^ NO_2_^−^ + O_2_ reaction pathway. Bond lengths are given in angstrom.

As indicated in Figure 4, the mechanism of the NO + O_2_^−^ reaction takes place by an immediate head-on-head addition reaction. In this mechanism, an oxygen atom from the superoxide species migrates to the neighboring NO molecule forming the transition state TS4. In TS4, the breaking O-O bonds are stretched by 69% (2.105 Å) compared to the equilibrium lengths in O_2_^−^, while the N-O bond being formed is 6% (1.24 Å) shorter than the free NO_2_^−^. The O atom located in O_2_^−^ associates with N in NO, and the dissociation of O-O bond takes place at the same instant, thus resulting in the formation of NO_2_^−^ and singlet oxygen. The activation barrier for the reaction, as shown in Scheme 4, is 1.27 kcal/mol, which indicates that the reaction could take place easily at room temperature. Taking into account the atmospheric conditions: the reaction takes place as barrierless. The experimental data available for the formation of NO_2_^-^ through the binary reaction of NO and O_2_^−^ reported by Stariket et al. [30] shows a reaction barrier of 0.0 kcal/mol, which goes hand-in-hand with our observation. The association reaction of O_2_^−^ with NO is found to be exergonic by −24.21 kcal/mol.

**5.** N_2_ + O_3_^− →^ NO_2_^−^ + NO

**Figure 5:**
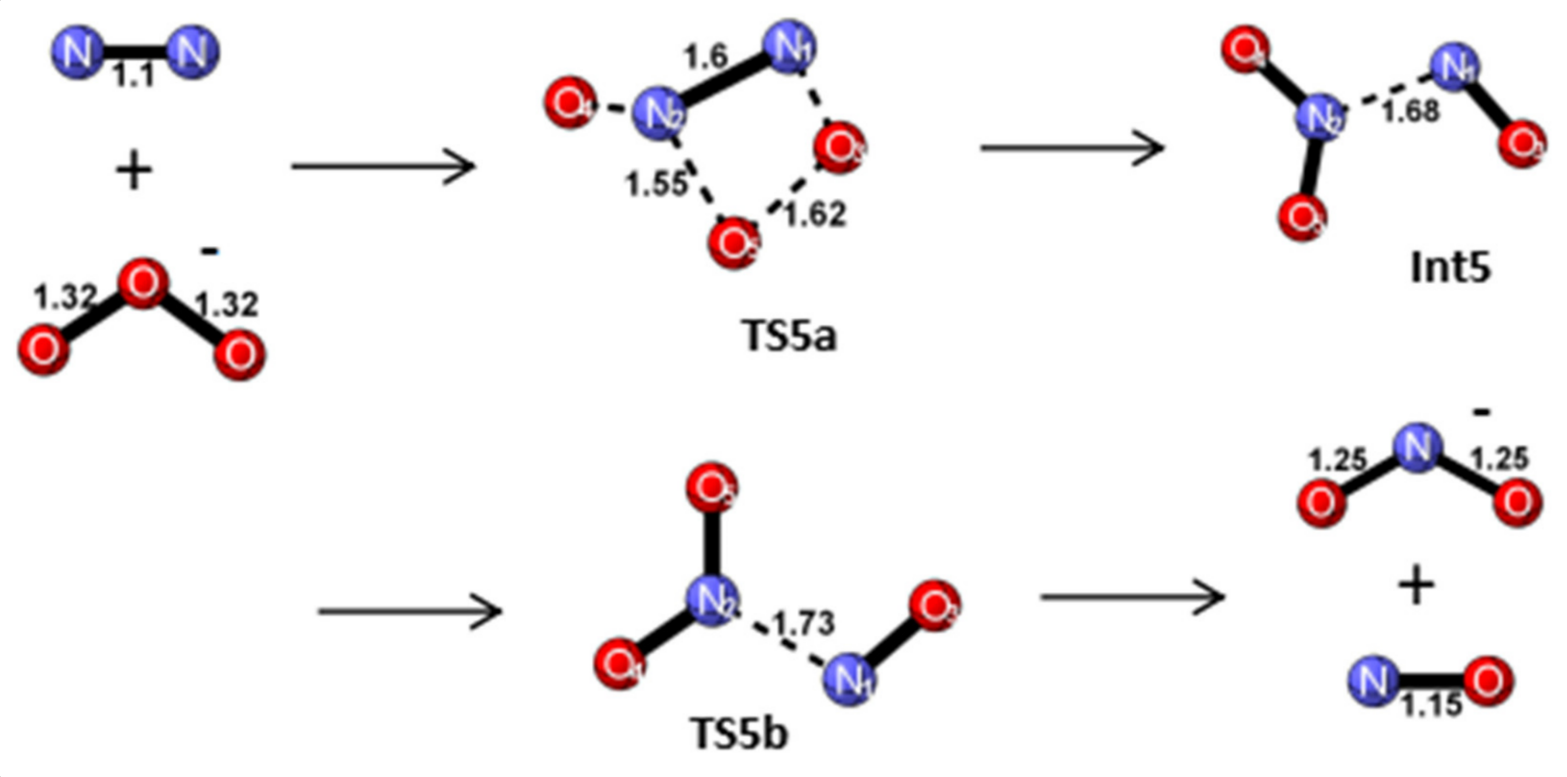
Optimized geometries of the reactants, transition state and products along N_2_ + O_3_^− →^ NO_2_^−^ + NO reaction pathway. Bond lengths are given in angstrom.

The reaction of O_3_^−^ with N_2_ forming NO_2_^−^ and NO as the products was found to take place in two steps with an intermediate. The reaction is unachievable at room temperature since it requires high energy to break the strong triple bonds between the two nitrogen atoms. However, it could occur at elevated temperatures, such as in jet engine combustion processes or in the ionosphere region of the atmosphere, where the average temperature range is 200–1500 °C. The previous work by Starik et al. [30] experimentally reported the formation of these products. For analyzing the mechanism of this uncommon reaction and identifying the transient species included, a PES calculation was initially performed at the CCSD level of theory by varying the N-O bond distance between 1 Å to 2 Å. Two transition points were located at the N-O bond length of 1.6 Å and 1.2 Å. The geometry obtained at these points was optimized, and an adduct-like transition state (TS5a) having an imaginary frequency of −1687.3 cm^−1^ and a corresponding N-O bond distance of 1.55 Å was obtained. The calculated activation barrier for the reaction is 27.1 kcal/mol at the CCSD(T)/aug-cc-pVTZ level of theory, as shown in Scheme 5, which further confirms that the reaction is feasible only at high-temperature conditions. Optimizing the second transition point obtained at 1.2 Å gave a transition state with imaginary frequency −247.8 cm^−1^. As shown in Figure 5, the reaction initiates by the attack of both of the N atoms of N_2_ with all three oxygen atoms of O_3_^−^ forming an adduct through TS5a, where corresponding O4-O5, O5-O3, N1-N2 bond elongates by 0.9, 0.3 and 0.5 Å, respectively, from the optimized geometry. Subsequently, TS5a converts to Int5 by the cleavage of the O5-O3 bond and the further elongation of the N1-N2 bond. From the intermediate, products NO_2_^−^ and NO is formed instantaneously through non-planar transition state TS5b, having an N1-N2 bond length of 1.73 Å and a barrier height of 3.2 kcal mol^−1^. The highly polar NO_2_^−^ could react with the H^+^/H_3_O^+^ in the atmosphere to form nitric acid. Additionally, the NO formed in the reaction could further react with the atmospheric aerosols and generate more complicated reactive clusters with severe negative impacts.

## 4. Conclusions

In this study, we used CCSD calculations to investigate the atmospheric association reaction mechanisms of N_2_/NO_x_ with the O_n_^−^ (*n* = 2, 3) chemi-ions. The computed geometrical parameters obtained with CCSD reasonably agree with the previous experimentally measured values. Our calculations reveal that two of these gas-phase reactions occur with the involvement of intermediate structures, while the other three occur in a concerted manner. The transition states involved in the reactions were identified and characterized. Among the reactions considered in this work, the binary association reaction of O_3_^−^ with NO could take place via two reaction pathways, forming two different products. Out of these two reactions, the formation of NO_2_^−^ by the direct attachment of terminal oxygen of O_3_^−^ with NO is about 8.47 kcal/mol energetically more favourable than the formation of NO_3_^−^, which authenticates previous experimental studies. The probable reaction channel for the formation of NO_3_^−^ from NO and O_3_^−^ starts with the addition of O_3_^−^ on NO, forming a four-membered cyclic transition state, subsequently forming an intermediate, which then dissociates into the product through a transition state with *C_s_* symmetry. NO_3_^−^ could also be furnished via the combination of NO_2_ with O_3_^−^ through a one-step, concerted reaction with a single transition state. In the reaction between NO and O_2_^−^, the direct dissociation of the oxygen atom leads to the formation of NO_2_^−^ as the product. The reaction has an activation barrier of 1.27 kcal/mol, which could be considered a barrierless process in the atmosphere. The reaction between N_2_ and O_3_^−^ is a two-step process where an adduct-like intermediate is formed through a transition state with *C*_1_ symmetry. The product NO_2_^−^ is formed by the dissociation of the intermediate. The reaction has a significantly high activation barrier of 27.1 kcal/mol for this reaction, which suggests its unfeasibility under normal reaction conditions. The thermochemical analysis of the model reactions shows that the reaction between N_2_ and O_3_ could take place under the general atmospheric condition. The reaction of molecular nitrogen with O_3_^−^ usually occurs in regions with high temperature, such as the ionosphere region of the atmosphere or in jet engine exhaust, where the temperature range on average is between 200–1500 °C.

The main products in all the reactions considered here are a series of exceptionally reactive and highly polar nitrogen oxide anions, which could either get neutralized via ion-ion recombination or interact with different molecules in the atmosphere to form large cluster ions. Moreover, the by-products in these reactions involve very reactive free radicals, which could be consumed by other atmospheric reactive species and contribute to the formation of secondary contaminants.

## Figures and Tables

**Table 1:** The optimized geometrical parameters for the species involved in the reactions N_2_/ NO_X_ + O_n_^− →^ NO_X_^−^ + O_n_: The bond lengths are given in angstrom, and the bond angles are given in degrees. Experimentally determined values are provided for comparison.

Molecule	Parameters	CCSD/ 6-31g(d)	CCSD/ 6-311g(d)	CCSD/ 6-311+g(d)	* Exp ^1,2,3^
NO	R(N-O)	1.166	1.144	1.151	1.151
O_2_	R(O-O)	1.220	1.200	1.204	1.207
N_2_	R(N-N)	1.102	1.102	1.103	1.098
O_2_^−^	R(O-O)	1.357	1.341	1.349	1.350
NO_2_	R(N-O)	1.204	1.190	1.198	1.207
	<O-N-O	134.3	134.5	134.1	134.1
NO_2_^−^	R(N-O)	1.271	1.254	1.253	1.257
	<O-N-O	115.7	116.1	116.1	116.0
NO_3_^−^	R(N-O)	1.261	1.251	1.268	1.270
	<O-N-O	120.0	120	120.0	120.0
O_3_^−^	R(O-O)	1.36	1.342	1.319	1.314
	<O-O-O	115.3	115.4	114.6	114.6

^1^ [55] ^2^ [56] ^3^ [57]. * Exp—Experimental values.

**Table 2:** The vibrational frequencies of the reactants and products for the reaction N_2_/NO_X_ + O_n_^− →^ NO_x_^−^ + O_n_ using different levels of CCSD theory. Frequencies are given in cm^−1^. The experimentally determined values are provided for comparison.

Molecule	CCSD/6-31g(d)	CCSD/ 6-311g(d)	CCSD/ 6-311+g(d)	Exp ^1,2,3,4^
NO	1951.7	1971.9	1963.8	1904.1
O_2_	1650.1	1678.9	1667.0	1580.4
N_2_	2411.12	2411.1	2408.0	2330.0
O_2_^−^	1208.0	1214.5	1183.5	1074.0
NO_2_	770.1	713.7	784.0	756.8
	1352.4	1226.4	1375.0	1355.9
	1383.3	2181.6	2106.1	1663.5
NO_2_^−^	801.9	822.1	814.9	776.0
	1386.8	1385.1	1353.9	1241.5
	1402.8	1414.4	1381.2	1284.0
NO_3_^−^	715.3	735.8	725.0	720.0
	860.1	875.8	852.0	830.0
	1099.9	1109.0	1101.4	1050.0
	1503.8	1493.6	1427.0	1390.0
O_3_^−^	590.3	617.2	671.6	-
	1006.7	984.7	1102.9	-
	1006.8	1089.3	1321.1	-

^1^ [58] ^2^ [55] ^3^ [56] ^4^ [57].

**Table 3:** Calculated total Energy and zero-point energies of reactants and products for the reactions N_2_/NO_X_ + O_n_^− →^ NO_3_^−^ + O at different levels of CCSD theory. Energy and zero-point energy (ZPE) are given in atomic units.

Molecule	Parameters	CCSD/ 6-31G(d)	CCSD/ 6-311G(d)	CCSD/ 6-311+G(d)
NO	Energy	−129.584286	−129.641792	−129.647432
	Z.P.E	0.004446	0.004492	0.004474
O_2_	Energy	−149.967988	−150.041012	−150.047750
	Z.P.E	0.003759	0.003825	0.003798
N_2_	Energy	−109.308796	−109.308790	−109.313030
	Z.P.E	0.005493	0.005493	0.005487
O_2_^−^	Energy	−149.936820	−150.010960	−150.048378
	Z.P.E	0.002752	0.002764	0.002696
NO_2_	Energy	−204.565748	−204.638166	−204.664268
	Z.P.E	0.007987	0.009390	0.013591
NO_2_^−^	Energy	−204.661106	−204.704836	−204.744319
	Z.P.E	0.008182	0.008251	0.008088
NO_3_^−^	Energy	−279.628433	−279.759869	−279.78294
	Z.P.E	0.014577	0.014680	0.014261
O_3_^−^	Energy	−224.912758	−225.020595	−225.060074
	Z.P.E	0.006069	0.006131	0.005914

## Data Availability

Not applicable.

## Supplementary Materials

The following supporting information can be downloaded at: https://www.mdpi.com/article/10.3390/e24091257/s1, Table S1: Vibrational Analysis.
