# Supplementary material for: A Quantum Chemical Investigation into the Molecular Mechanism of the Atmospheric Reactions of Chemi-Ions with Nitrogen and Nitrogen Oxides
Source: Entropy (Basel). 2022 Sep 7;24(9):1257. doi: 10.3390/e24091257 (PMC9497496; doi:10.3390/e24091257)
Supplement: Supplementary file 1 [file entropy-24-01257-s001.zip › entropy-1859342-supplementary.pdf]

A Quantum Chemical Investigation into the Molecular Mechanism of the Atmospheric Reactions of Chemi-ions with Nitrogen and Nitrogen Oxides.

RehinSulay[1], AnandhuKrishnan[1], BalasubramoniamMuraliKrishna[1], ChandralekhaRajalakshmi[1], Sudheesh Devadas[1], Jintumol Mathew[1], VibinIpeThomas[1,2,*]

[1]*Departmentof Chemistry, CMS College Kottayam (Autonomous), Kottayam, Kerala, India,686001.*

[2]*Institute for Integrated Programmes and Research in Basic Sciences, Mahatma Gandhi University, Priyadarsini Hills P.O, Kottayam, Kerala, India, 686560.*

E-mail: vibin@cmscollege.ac.in Ph: (+91) 82 899 18958

Reference

Gaussian 09, Revision *D.01*, M. J. Frisch, G. W. Trucks, H. B. Schlegel, G. E. Scuseria, M. A. Robb, J. R. Cheeseman, G. Scalmani, V. Barone, B. Mennucci, G. A. Petersson, H. Nakatsuji, M. Caricato, X. Li, H. P. Hratchian, A. F. Izmaylov, J. Bloino, G. Zheng, J. L. Sonnenberg, M. Hada, M. Ehara, K. Toyota, R. Fukuda, J. Hasegawa, M. Ishida, T. Nakajima, Y. Honda, O. Kitao, H. Nakai, T. Vreven, J. A. Montgomery, Jr., J. E. Peralta, F. Ogliaro, M. Bearpark, J. J. Heyd, E. Brothers, K. N. Kudin, V. N. Staroverov, R. Kobayashi, J. Normand, K. Raghavachari, A. Rendell, J. C. Burant, S. S. Iyengar, J. Tomasi, M. Cossi, N. Rega, J. M. Millam, M. Klene, J. E. Knox, J. B. Cross, V. Bakken, C. Adamo, J. Jaramillo, R. Gomperts, R. E. Stratmann, O. Yazyev, A. J. Austin, R. Cammi, C. Pomelli, J. W. Ochterski, R. L. Martin, K. Morokuma, V. G. Zakrzewski, G. A. Voth, P. Salvador, J. J. Dannenberg, S. Dapprich, A. D. Daniels, O. Farkas, J. B. Foresman, J. V. Ortiz, J. Cioslowski, and D. J. Fox, Gaussian, Inc., Wallingford CT, 2016.

Vibrational analysis

Molecule	CCSD/6-31g(d)	CCSD/ 6-311g(d)	CCSD/ 6-311+g(d)	Exp[1,2,3,4]	Modes
NO	1951.7	1971.9	1963.8	1904.1	Stretching
O_2	1650.1	1678.9	1667.0	1580.4	Stretching
N_2	2411.12	2411.1	2408.0	2330.0	Stretching
O_2^-	1208.0	1214.5	1183.5	1074.0	Stretching
NO_2	770.1	713.7	784.0	756.8	In-plane bending
	1352.4	1226.4	1375.0	1355.9	Symmetric stretching
	1383.3	2181.6	2106.1	1663.5	Asymmetric stretching
NO_2^-	801.9	822.1	814.9	776.0	In-plane bending
	1386.8	1385.1	1353.9	1241.5	Asymmetric stretching
	1402.8	1414.4	1381.2	1284.0	Symmetric stretching
NO_3^-	715.3	735.8	725.0	720.0	In-plane bending
	860.1	875.8	852.0	830.0	Out-of-plane bending
	1099.9	1109.0	1101.4	1050.0	Symmetric stretching
	1503.8	1493.6	1427.0	1390.0	Asymmetric stretching
O_3^-	590.3	617.2	671.6	a	
	1006.7	984.7	1102.9	a	
	1006.8	1089.3	1321.1	a	

a No available experimental data.

Table S1: The vibrational frequencies of the reactants and products for the reaction: $N_2/NO_x + O_n^- \rightarrow NO_x^- + O_n$using different levels of CCSD theory. Frequencies are given in cm^{-1}. Experimentally determined values are provided for comparison.

CARTESIAN COORDINATES OF ALL THE TRANSITION STATES INVOLVED IN THE REACTION

TS1

O	-0.48602000	-1.07154200	-0.15114600
O	-1.39729300	0.00009400	0.08374200

O	-0.48578200	1.07153100	-0.15116300
O	1.70173800	-0.00006900	-0.20934100
N	0.76269400	-0.00001600	0.48903800

TS2a

N	0.30276400	0.00000300	0.00001800
O	0.88759400	-1.09895200	0.00005100
O	-1.02004900	0.00006200	0.74992900
O	-1.02001800	-0.00008500	-0.74994600
O	0.88755600	1.09897200	-0.00004900

TS2b

N	-0.49590700	-0.00000100	0.07563100
O	-1.00479300	-1.07897300	-0.14807800
O	1.70429000	-0.00000200	-0.45052600
O	0.73923100	0.00001300	0.68051400
O	-1.00480900	1.07896300	-0.14808800

TS3

N	-2.95682000	1.92008900	0.92126300
O	-3.77168000	1.02302800	0.99982800
O	-3.16036500	3.04767300	0.54066600
O	-0.57019500	2.66518300	1.56979400
O	-1.12021300	1.53956700	1.29046000
O	-0.70067300	3.02842000	2.86578100

TS4

N	-0.66125700	0.35057800	0.40135200
O	-1.32857500	-0.43483400	-0.18797200
O	1.37377700	-0.52736100	0.08275200
O	0.53339900	0.65543900	-0.24596200S4

TS5a

O	1.34973900	0.03058800	-0.17974100

O	-1.64683200	0.12614700	-0.25632900
O	0.30177400	-1.16180500	0.02746600
N	-0.67580500	0.10976800	0.42409000
N	0.67044300	1.03196200	0.02101400

TS5b

N	-0.54187100	0.04485500	0.00016700
O	-0.54619600	1.26378100	0.00050000
O	-1.55027400	-0.65251300	0.00035500
N	0.87082100	-0.77591800	-0.00049300
O	1.80366000	0.03096200	-0.00053000
